# Quantitative assessment of the association between Fas/FasL gene polymorphism and susceptibility to esophageal carcinoma in a north Chinese population

**DOI:** 10.1002/cam4.633

**Published:** 2016-01-27

**Authors:** Meijuan Zhang, Cuiping Wu, Baohuan Li, Wenjun Du, Chuanzhen Zhang, Ziping Chen

**Affiliations:** ^1^ Department of GastroenterologyShandong Provincial Qianfoshan HospitalShandong UniversityJinanChina

**Keywords:** Esophageal carcinoma, Fas, FasL, polymorphism

## Abstract

The case–control study aims to investigate the association of Fas and FasL genetic polymorphisms (Fas‐670A/G (rs1800682), Fas‐1377G/A (rs2234767) and FasL‐844T/C (rs763110)) with esophageal carcinoma susceptibility in a north Chinese population. A total of 204 patients with esophageal carcinoma and 248 healthy controls were enrolled from Henan, China and genotyped by the polymerase chain reaction and restriction fragment length polymorphism method. There were no significant differences in distributions of their genotypes frequencies between patients and controls in Fas‐670A/G, Fas‐1377G/A and FasL‐844T/C polymorphisms (*P *>* *0.05). Stratified analysis showed that no significant association was found between esophageal carcinoma and gene polymorphisms of Fas‐670 A/G, Fas‐1377G/A, and FasL‐844T/C (*P *>* *0.05). Genetic polymorphisms in the death pathway genes Fas and FasL were not associated with risk of developing esophageal carcinoma in a north Chinese population.

## Introduction

Esophageal carcinoma is the eighth most common cancer worldwide and its incidence has significant regional and ethnic differences 1. China has a high incidence of esophageal carcinoma, and is one of the areas that have a relativity high fatality rate 2. But its definite etiology and pathogenesis are not clear yet. Long‐term stimulations from certain physicochemical factors and carcinogens from food are one of the major pathogens, and genetic factors also participate in the occurrence of esophageal carcinoma 3. Significant familial aggregation is found in the development of esophageal carcinoma and lots of related genes changes are discovered in some high incidence cancer families. Recent years, research on genetic susceptibility to cancer research has increasingly become a hot spot.

Human Fas is located on 10q24.1 chromosome, encoding transmembrane protein I with 319 amimo acid residual and 43KD of molecular weight 4. Human FasL is located at 23 region in number 1 chromosome and it encodes transmembrane protein II with 281 amimo acid residual and 40KD of molecular weight 5. In the known pathways of apoptosis, Fas/FasL signaling pathway has an important biological effect. Fas, also known as CD95, TNFSF6, or APO‐1, is a cell surface receptor involved in apoptotic signal transmission in many cell types and interacts with its natural ligand (known as FasL, CD95L) to initiate the death signal cascade that leads to apoptotic cell death 4, 6. Reduced Fas expression and (or) increased FasL expression conducive to tumor development and progression 7. In addition, somatic mutations and functional germline in gene Fas and FasL impair apoptotic signal transduction, which are related to a high risk of cancer 8, 9, 10. Thus, Fas and FasL gene are approved to play an important role in the development and progression of cancer.

Previous studies revealed that the level of Fas expression was lower and the level of FasL was higher in esophageal carcinoma than that of the corresponding normal tissue 11, 12, 13. Besides, the down‐regulation of Fas may decrease the ability of undergoing apoptosis in esophageal carcinoma cells, while the up‐regulation of FasL increase the ability of counterattacking immune system by killing Fas‐sensitive lymphocytes 11, 12. In addition, aberrant expression of Fas and FasL was also related to differentiation, invasiveness, metastasis, and prognosis of cancer 11, 14.

Single‐nucleotide polymorphisms have been identified in the promoter region of the Fas gene, A or G at position‐670 (Fas‐670A/G) and G or A at position‐1377 (Fas‐1377G/A). The Fas‐670G allele and the Fas‐1377A allele disrupt STAT1and Sp1 transcription factor‐binding sites, respectively, and thus diminish promoter activity and decrease Fas gene expression 15, 16. The promoter of FasL also has a functional single‐nucleotide polymorphism, T or C at position‐844 (FasL‐844T/C), that is located in a binding modifier another transcription factor, CAAT/enhancer‐binding protein *β* 17. Higher basal expression of FasL is significantly associated with the FasL‐844 C allele than the FasL‐844 T allele 17.

To date, studies showed that the Fas‐670A/G, Fas‐1377G/A, and FasL‐844T/C polymorphisms might be associated with increased risk of certain cancers, including breast cancer 18, 19, 20, 21, gastric cancer 22, 23, cervical cancer 24, 25, lung cancer 26, 27, etc. Several studies have reported the potential association between Fas/ FasL polymorphisms and risk of esophageal cancer 28, 29. However, the results were not always consistent with one another, partially because of different sample sizes, ethnic backgrounds, and publication bias. In the case–control study, we aim to identify the genotyping of Fas‐670A/G, Fas‐1377G/A, FasL‐844T/C in all cases, and to explore the correlation between three polymorphisms and susceptibility of esophageal carcinoma in north China.

## Methods

### Study participants

From 2005 January to 2006 December, two hundred and four participants with esophageal carcinoma at Anyang Tumor Hospital (Henan Province, China) were recruited. All esophageal carcinoma patients were diagnosed according to histopathology. Patients with any previous cancers or autoimmune diseases, chemotherapy or radiotherapy and incomplete clinical information were excluded from the study. Demographic information, including age, sex, tobacco smoking, and alcohol drinking history were obtained from medical records.

Two hundred and forty eight healthy blood donors were selected as control group in Henan Anyang. Criteria for donor eligibility were as follows: (1) permanent residents in rural Anyang; (2) age between 25 to 80 years; (3) no self‐reported history of cancer, previous cardiocerebral vascular diagnoses or psychological disorders; (4) no self‐reported history of infection with hepatitis B virus, hepatitis C virus, or human immunodeficiency virus (and no evidence of these infections based on blood screening).

Each participant was required to sign an informed consent and complete a questionnaire in one‐on‐one interviews. After the interview, 5‐mL samples of venous blood were collected from each subject. We defined regular cigarette smoking as a history of at least 1 cigarette per day for ≥12 months or ≥18 packs for 1 year, and regular alcohol consumption was defined as drinking Chinese liquor at least twice per week for ≥12 months (other kinds of regular drinker such as beer and red wine is very rare in local area). The study protocol was approved by the Ethics Committees of Shandong Provincial Qianfoshan Hospital.

### Genotyping

Genome DNA was extracted from peripheral blood of research objects, using the QIAGEN DNA Isolation Kit (QIAGEN, Dusseldorf, Germany). Polymerase chain reaction‐restriction fragment length polymorphism (PCR‐RFLP) methods were performed for genotyping Fas‐670A/G, Fas‐1377G/A, FasL‐844T/C. Information of primers sequences, PCR products sizes, restriction enzymes, enzyme digestion temperatures and restriction products are shown in Table S1. PCR was performed with 30 *μ*L of reaction mixture containing 50–100 ng genomic DNA, 0.5 *μ*L dNTPs (10 mmol/L, Invitrogen, Carlsbad, CA, USA), 0.5 *μ*L each primer (10 mmol/L, Atobo BioTECH Co., Ltd, Shanghai, China), 0.5 U Taq DNA polymerase (5 U/*μ*L, KeyGEN BioTECH Co., Ltd, Nanjing, China) and 3 *μ*L 10 × PCR buffer. The PCR reaction mixture was initially denatured at 94°C for 2 min, followed by 36 cycles of 30 sec at 94°C, 30 sec at denaturation temperature, 60 sec at 72°C, and a final extension of 5 min at 72°C. Suitable primers for each polymorphism were used to amplify the corresponding PCR products, and restriction products were digested by the appropriate restriction enzymes. Restriction DNA products were separated by 2% agarose gel electrophoresis and visualized by ultraviolet light. In addition, a random 10% of samples were selected for confirmation by Sanger sequencing.

### Statistical analysis

All statistical analyses were conducted, using SPSS software (version 13.0 for Windows, Chicago, IL). Mann–Whitney *U* test was applied to compare the age distribution between case and control group. Chi‐Square test was used to analyze the gender, smoking status, and drinking status discrepancy between case and control group. Hardy–Weinberg balance test was used to verify the crowd representativeness of the research sample, count the genotype frequencies and allelic gene frequencies. The associations of esophageal carcinoma risk with genotypes were analyzed, using an unconditional logistic regression model for adjusted odds ratio (OR) when adjusting for age, sex, tobacco smoking, and alcohol drinking. Then, OR and its 95% confidence intervals (95% CI) were counted to assess the correlation between genotypes and susceptibility to esophageal carcinoma.

## Results

### Demographics of the enrolled subjects

The distribution of demographic parameters between esophageal cancer patients and controls was shown in Table**** 1. The mean age was 50.58 ± 7.55 years for patients and 49.58 ± 8.44 years for controls, and 63.73% and 36.27% of the patients and 66.13% and 33.87% of the controls were men and women, respectively. No significant differences were found between patients and control subjects in terms of age, gender, smoking status, and drinking status (*P *=* *0.074, *P *=* *0.594, *P *=* *0.099 and *P *=* *0.487), suggesting that the frequency matching was adequate.

**Table 1:** Distribution of demographic characteristics in patients with esophageal carcinoma and controls

Characteristics	Patients (*n* = 204)		Controls (*n* = 248)		*P*
	*n*	%	*n*	%	
Age (year, mean ± SD)	50.58 ± 7.55		49.58 ± 8.44		0.074^a^
Gender					
Male	130	63.73	164	66.13	0.594^b^
Female	74	36.27	84	33.87	
Tobacco smoking status					
Ever	93	45.59	94	37.90	0.099^b^
Never	111	54.41	154	62.10	
Alcohol consumption status					
Ever	62	30.39	68	27.42	0.487^b^
Never	142	69.61	180	72.58	

a Mann–Whitney *U* test.

b *c* ^*2*^ test.

### Gene polymorphisms and ESCC risk

Distributions of the genotypes frequencies of the three polymorphisms among patients and controls are shown in Table**** 2. Fas‐670A/G, Fas‐1377G/A and FasL‐844T/C showed polymorphism in all research subjects. And the genotype distributions of Fas‐670A/G, Fas‐1377G/A and FasL‐844T/C in two groups were consistent with Hardy–Weinberg equilibrium (*P *>* *0.05). For Fas‐670A/G polymorphism, the frequencies of GG, AG, and AA genotypes were 12.75%, 50.98%, and 36.27% among the patients and 16.53%, 47.98%, and 35.48% among the controls. The frequencies of AA, AG, and GG genotypes for Fas‐1377G/A were 13.23%, 45.10% and 41.67% among the patients and 13.71%, 45.97% and 40.32% among the controls. For FasL‐844T/C, the frequency of genotype TT, TC, and CC in the esophageal carcinoma patients and in the healthy controls was 3.92%, 39.11%, 56.45% and 4.44%, 39.11%, 56.45%, respectively. Distributions of these Fas and FasL genotypes were then compared among patients and control subjects (*P *>* *0.05). Frequencies of Fas‐670A/G, Fas‐1377G/A and FasL‐844T/C genotypes among case patients did not differ statistically significantly from those among control subjects. Logistic regression analysis indicated that there was no significant association between esophageal carcinoma and gene polymorphisms of Fas‐670A/G, Fas‐1377G/A and FasL‐844 T/C (Fas‐670AG, *P *=* *0.820; Fas‐670GG, *P *=* *0.451; Fas‐1377AG, *P *=* *0.897; Fas‐1377AA, *P *=* *0.881; FasL‐844TC, *P *=* *0.119; FasL‐844CC, *P *=* *0.454).

**Table 2:** Distribution of genotypes and alleles of Fas/FasL SNPs between esophageal carcinoma patients and controls

Genotype	Patients (*n* = 427)		Controls (*n* = 427)		OR (95% CI)^a^	*P* value^b^
	*n*	%	*n*	%		
Fas‐670A/G						
AA	74	36.27	88	35.48	1.000	–
AG	104	50.98	119	47.98	1.049 (0.696–1.581)	0.820
GG	26	12.75	41	16.53	0.797 (0.442–1.438)	0.451
Fas‐1377G/A						
GG	85	41.67	100	40.32	1.000	–
AG	92	45.10	114	45.97	0.974 (0.650–1.458)	0.897
AA	27	13.23	34	13.71	0.956 (0.529–5.671)	0.881
FasL‐844T/C						
CC	103	56.45	140	56.45	1.000	–
TC	93	39.11	97	39.11	1.012 (0.393–2.604)	0.981
TT	8	3.92	11	4.44	0.767 (0.524–1.124)	0.174

a Odds ratios (OR), Confidence intervals (CI).

b *P* value adjusted for age, gender, tobacco smoking, and alcohol consumption status.

### Stratification analysis of polymorphisms and ESCC risk

To evaluate the effects of Fas and FasL genotypes on the risk of esophageal carcinoma, patients and controls were stratified based on age, sex, smoking status, and drinking status (Table**** 3). The results showed that no significant association was found between esophageal carcinoma and gene polymorphisms of Fas‐670A/G, Fas‐1377G/A, and FasL‐844T/C in the north Chinese population (*P *>* *0.05).

**Table 3:** Stratified analysis between Fas and FasL polymorphisms and esophageal carcinoma risk by selected status

Variable	Fas‐670A/G (case/control)			*P* a; Adjusted OR^a^ (95% CI)			Fas‐1377G/A (case/control)			*P* a; Adjusted OR^a^ (95% CI)		
	AA	AG	GG	AA	AG	GG	GG	AG	AA	GG	AG	AA
Sex												
Male	48/57	66/77	16/30	1.000	0.951	0.311	54/64	59/75	17/26	1.000	0.509	0.748
	–	–	–	–	1.016 (0.609–1.696)	0.685 (0.329–1.425)	–	–	–	–	0.783 (0.379–1.617)	0.921 (0.555–1.525)
Female	26/31	38/40	10/11	1.000	0.635	0.970	31/37	33/37	10/8	1.000	0.540	0.779
	–	–	–	–	1.183 (0.592–2.364)	1.020 (0.364–2.857)	–	–	–	–	1.398 (0.478–4.086)	1.101 (0.562–2.157)
Age												
≤60	32/51	50/62	14/16	1.000	0.393	0.265	39/56	43/62	11/14	1.000	0.084	0.939
	–	–	–	–	1.291 (0.718–2.320)	1.641 (0.687–3.921)	–	–	–	–	2.261 (0.895–5.709)	1.023 (0.577–1.812)
>60	42/37	54/57	12/25	1.000	0.588	0.060	46/44	49/52	13/23	1.000	0.138	0.830
	–	–	–	–	0.849 (0.470–1.535)	0.449 (0.195–1.034)	–	–	–	–	0.540 (0.239–1.220)	0.938 (0.524–1.680)
Tobacco smoking												
Ever	32/36	52/45	9/13	1.000	0.263	0.424	38/38	45/42	10/14	1.000	0.277	0.661
	–	–	–	–	1.438 (0.761–2.715)	0.661 (0.240–1.824)	–	–	–	–	0.584 (0.222–1.540)	1.151 (0.613–2.161)
Never	42/52	52/74	17/28	1.000	0.529	0.553	47/62	47/72	17/20	1.000	0.639	0.566
	–	–	–	–	0.839 (0.485–1.450)	0.800 (0.383–1.672)	–	–	–	–	1.200 (0.560–2.572)	0.855 (0.501–1.459)
Alcohol consumption												
Ever	22/26	34/31	6/12	1.000	0.506	0.532	24/28	31/30	7/11	1.000	0.616	0.576
	–	–	–	–	1.310 (0.592–2.899)	0.682 (0.206–2.263)	–	–	–	–	0.743 (0.232–2.374)	0.878 (0.545–1.414)
Never	52/62	79/88	20/29	1.000	0.860	0.692	61/72	61/84	20/23	1.000	0.803	0.592
	–	–	–	–	0.957 (0.589–1.557)	0.870 (0.438–1.729)	–	–	–	–	1.093 (0.543–2.199)	1.252 (0.569–2.754)

Variable	FasL‐844T/C (case/control)		*P* a; Adjusted OR^a^ (95% CI)	
	CC	TT + TC	CC	TT+TC
Sex				
Male	66/94	64/70	1.000	0.263
	–	–	–	0.768 (0.484–1.220)
Female	37/47	37/37	1.000	0.446
	–	–	–	0.783 (0.416–1.471)
Age				
≤60	45/66	51/63	1.000	0.525
				0.842 (0.496–1.430)
>60	58/74	50/45	1.000	0.196
	–	–	–	0.705 (0.415–1.198)
Tobacco smoking				
Ever	45/50	48/44	1.000	0.511
	–	–	–	0.825 (0.465–1.465)
Never	58/90	53/64	1.000	0.317
	–	–	–	0.778 (0.476–1.272)
Alcohol Consumption				
Ever	27/40	35/29	1.000	0.100
	–	–	–	0.559 (0.280–1.119)
Never	76/100	66/79	1.000	0.675
	–	–	–	0.910 (0.584–1.416)

a Adjusted for age, sex, tobacco smoking, alcohol consumption (besides stratified factors accordingly) in conditional logistic regression model.

## Discussion

Esophageal carcinoma is one of the most common malignant cancers of the digestive tract, especially in China. Among the main causes of esophageal cancer, genetic aberration plays a key role. The case–control study was conducted to investigate the relationship between polymorphisms in Fas‐670A/G, Fas‐1377G/A, and FasL‐844T/C and the susceptibility to esophageal carcinoma in Anyang, a north Chinese district with a high incidence of esophageal cancer.

Since the identification of polymorphisms in gene Fas and FasL, a variety of case–control studies have been published to explore the possible association between Fas‐670A/G, Fas‐1377G/A, and FasL‐844T/C and risk of cancer 18, 23, 24, 27; however, the reported results were conflicting. In our study, no significant association was found between polymorphisms Fas‐670A/G, Fas‐1377G/A, and FasL‐844T/C and susceptibility to esophageal cancer in Henan Anyang (*P *>* *0.05), suggesting that these polymorphisms might not play an important role in the progression and development of esophageal cancer in this particular population. These results are consistent with published report by Jain M et al. in India's population and Chen XB et al. in the Mongolian population 30, 31. In another study by Sun et al., subjects with Fas‐670GG (OR = 1.72, 95% CI = 1.26–2.34, *P *<* *0.001), Fas‐1377AA (OR = 1.79, 95% CI = 1.29–2.48, *P *<* *0.001) and FasL‐844CC (OR = 2.06, 95% CI = 1.64–2.59, *P *<* *0.001) genotypes were associated with increased risk of esophageal carcinoma compared with those with Fas‐670 AA, Fas‐1377 GG, FasL‐844 TT genotypes, respectively 29. The frequency of the polymorphisms Fas‐670A/G, Fas‐1377G/A, and FasL‐844 T/C in our study did not show statistical significance when compared to patients and controls. We deduced that the difference in sample size and sample sources between cases and controls might account for this inconsistency. In Sun's study, esophageal cancer patients were selected from the Cancer Hospital, Chinese Academy of Medical Sciences, and controls were enrolled from a nutritional survey database conducted in Beijing and the surrounding regions. As we knew, most of these patients came from other regions of China and they have different genetic backgrounds. Different levels of environmental exposure may affect the carcinogenesis progression in population with different genetic backgrounds. Considering geographical variation in the incidence of esophageal cancer, the matching mode might reduce the comparative efficiency between cases and controls, and even lead to a result of deviation. Conversely, the population in our study was mostly from the same area of high incidence of esophageal cancer and the study participants were relatively homogeneous in terms of genetic background and environmental risk factors. Moreover, previous studies of Fas and FasL genetic polymorphism‐related susceptibility to various cancers in China revealed that the frequency of genotypes varied in different populations even in the same country 23, 32, 33.

Several limitations of this case–control study needs to be addressed. Firstly, the sample size determination was not based on power calculations, which might affect the accuracy of the results. Secondly, the lack of a prior power calculation might increase the false positive rate in regression models. Besides, the sample size was relatively small which might weaken the statistical power of our study. Finally, participants in our study were restricted to a north Chinese population. Since the role of genetic polymorphism in tumor risk may be different with different ethnic populations, future researches of other ethnicities are needed.

In conclusion, we have shown that the Fas‐670A/G, Fas‐1377G/A, and FasL‐844T/C polymorphisms were not significantly associated with risk of esophageal cancer in a north Chinese population. These findings suggest that the promoter polymorphisms of the Fas and FasL genes may not contribute to the pathogenesis of esophageal cancer.

## Conflict of interest

None declared.

## Supporting information


**Table S1.** Supplementary Table 1 PCR and RFLP procedures and expected products of three polymorphisms in Fas/FasL gene.

## References

[1] Enzinger, P. C. , and R. J. Mayer . 2003 Esophageal cancer. N. Engl. J. Med. 349:2241–2252. 14657432 10.1056/NEJMra035010

[2] Liu, M. , M. Su , D. P. Tian , G. H. Zhang , H. L. Yang , and Y. X. Gao . 2010 Heredity, diet and lifestyle as determining risk factors for the esophageal cancer on Nanao Island in Southern China. Fam. Cancer 9:229–238. 19916058 10.1007/s10689-009-9300-6

[3] Wu, C. , Z. Hu , Z. He , W. Jia , F. Wang , Y. Zhou , et al. 2011 Genome‐wide association study identifies three new susceptibility loci for esophageal squamous‐cell carcinoma in Chinese populations. Nat. Genet. 43:679–684. 21642993 10.1038/ng.849

[4] Itoh, N. , S. Yonehara , A. Ishii , M. Yonehara , S. Mizushima , M. Sameshima , et al. 1991 The polypeptide encoded by the cDNA for human cell surface antigen Fas can mediate apoptosis. Cell 66:233–243. 1713127 10.1016/0092-8674(91)90614-5

[5] Suda, T. , T. Takahashi , P. Golstein , and S. Nagata . 1993 Molecular cloning and expression of the Fas ligand, a novel member of the tumor necrosis factor family. Cell 75:1169–1178. 7505205 10.1016/0092-8674(93)90326-l

[6] Oehm, A. , I. Behrmann , W. Falk , M. Pawlita , G. Maier , C. Klas , et al. 1992 Purification and molecular cloning of the APO‐1 cell surface antigen, a member of the tumor necrosis factor/nerve growth factor receptor superfamily. Sequence identity with the Fas antigen. J. Biol. Chem. 267:10709–10715. 1375228

[7] Reichmann, E. 2002 The biological role of the Fas/FasL system during tumor formation and progression. Semin. Cancer Biol. 12:309–315. 12147205 10.1016/s1044-579x(02)00017-2

[8] Davidson, W. F. , T. Giese , and T. N. Fredrickson . 1998 Spontaneous development of plasmacytoid tumors in mice with defective Fas‐Fas ligand interactions. J. Exp. Med. 187:1825–1838. 9607923 10.1084/jem.187.11.1825 PMC2212316

[9] Lee, S. H. , M. S. Shin , W. S. Park , S. Y. Kim , S. M. Dong , J. H. Pi , et al. 1999 Alterations of Fas (APO‐1/CD95) gene in transitional cell carcinomas of urinary bladder. Cancer Res. 59:3068–3072. 10397246

[10] Lee, S. H. , M. S. Shin , W. S. Park , S. Y. Kim , H. S. Kim , J. Y. Han , et al. 1999 Alterations of Fas (APO‐1/CD95) gene in non‐small cell lung cancer. Oncogene 18:3754–3760. 10391683 10.1038/sj.onc.1202769

[11] Bennett, M. W. , J. O'Connell , G. C. O'Sullivan , C. Brady , D. Roche , J. K. Collins , et al. 1998 The Fas counterattack in vivo: apoptotic depletion of tumor‐infiltrating lymphocytes associated with Fas ligand expression by human esophageal carcinoma. J. Immunol. 160:5669–5675. 9605174

[12] Gratas, C. , Y. Tohma , C. Barnas , P. Taniere , P. Hainaut , and H. Ohgaki . 1998 Up‐regulation of Fas (Apo‐1/CD95) ligand and down‐regulation of Fas expression in human esophageal cancer. Cancer Res. 58:2057–2062. 9605741

[13] Kase, S. , M. Osaki , H. Adachi , N. Kaibara , and H. Ito . 2002 Expression of Fas and Fas ligand in esophageal tissue mucosa and carcinomas. Int. J. Oncol. 20:291–297. 11788891

[14] Shibakita, M. , M. Tachibana , D. K. Dhar , T. Kotoh , S. Kinugasa , H. Kubota , et al. 1999 Prognostic significance of Fas and Fas ligand expressions in human esophageal cancer. Clin. Cancer Res. 5:2464–2469. 10499620

[15] Kurooka, M. , G. J. Nuovo , M. A. Caligiuri , and G. J. Nabel . 2002 Cellular localization and function of Fas ligand (CD95L) in tumors. Cancer Res. 62:1261–1265. 11888887

[16] Kordi Tamandani, D. M. , R. C. Sobti , and M. Shekari . 2007 Association of Fas‐670 gene polymorphism with risk of cervical cancer in North Indian population. Clin. Exp. Obstet. Gynecol. 35:183–186. 18754288

[17] Wu, J. , C. Metz , X. Xu , R. Abe , A. W. Gibson , J. C. Edberg , et al. 2003 A novel polymorphic CAAT/enhancer‐binding protein *β* element in the FasL gene promoter alters Fas ligand expression: a candidate background gene in African American systemic lupus erythematosus patients. J. Immunol. 170:132–138. 12496392 10.4049/jimmunol.170.1.132

[18] Hashemi, M. A. , S. Fazaeli , S. Ghavami , E. Eskandari‐Nasab , F. Arbabi , M. A. Mashhadi , et al. 2013 Functional polymorphisms of FAS and FASL gene and risk of breast cancer‐pilot study of 134 cases. PLoS ONE 8:e53075. 23326385 10.1371/journal.pone.0053075 PMC3543397

[19] Wang, W. , Z. Zheng , W. Yu , H. Lin , B. Cui , and F. Cao . 2012 Polymorphisms of the FAS and FASL genes and risk of breast cancer. Oncol. Lett. 3:625–628. 22740964 10.3892/ol.2011.541 PMC3362490

[20] Mahfoudh, W. , N. Bouaouina , S. Gabbouj , and L. Chouchane . 2012 FASL‐ 844 T/C polymorphism: A biomarker of good prognosis of breast cancer in the Tunisian population. Hum. Immunol. 73:932–938. 22732091 10.1016/j.humimm.2012.06.001

[21] Crew, K. D. , M. D. Gammon , M. B. Terry , F. F. Zhang , M. Agrawal , S. M. Eng , et al. 2007 Genetic polymorphisms in the apoptosis‐associated genes FAS and FASL and breast cancer risk. Carcinogenesis 28:2548–2551. 17962219 10.1093/carcin/bgm211

[22] Zhou, R. M. , N. Wang , Z. F. Chen , Y. N. Duan , D. L. Sun , and Y. Li . 2010 Polymorphisms in promoter region of FAS and FASL gene and risk of gastric cardiac adenocarcinoma. J. Gastroenterol. Hepatol. 25:555–561. 20074157 10.1111/j.1440-1746.2009.06116.x

[23] Wang, M. , D. Wu , M. Tan , W. Gong , H. Xue , H. Shen , et al. 2009 FAS and FAS ligand polymorphisms in the promoter regions and risk of gastric cancer in Southern China. Biochem. Genet. 47:559–568. 19565204 10.1007/s10528-009-9264-0

[24] Lai, H. C. , W. Y. Lin , Y. W. Lin , C. C. Chang , M. H. Yu , C. C. Chen , et al. 2005 Genetic polymorphisms of FAS and FASL (CD95/CD95L) genes in cervical carcinogenesis: an analysis of haplotype and gene‐gene interaction. Gynecol. Oncol. 99:113–118. 15996722 10.1016/j.ygyno.2005.05.010

[25] Lai, H. C. , H. K. Sytwu , C. A. Sun , M. H. Yu , C. P. Yu , H. S. Liu , et al. 2003 Single‐nucleotide polymorphism at Fas promoter is associated with cervical carcinogenesis. Int. J. Cancer 103:221–225. 12455036 10.1002/ijc.10800

[26] Park, S. H. , J. E. Choi , E. J. Kim , J. S. Jang , W. K. Lee , S. I. Cha , et al. 2006 Polymorphisms in the FAS and FASL genes and risk of lung cancer in a Korean population. Lung Cancer 54:303–308. 17014925 10.1016/j.lungcan.2006.09.002

[27] Zhang, X. , X. Miao , T. Sun , W. Tan , S. Qu , P. Xiong , et al. 2005 Functional polymorphisms in cell death pathway genes FAS and FASL contribute to risk of lung cancer. J. Med. Genet. 42:479–484. 15937082 10.1136/jmg.2004.030106 PMC1736067

[28] Zhao, H. , L. Zheng , X. Li , and L. Wang . 2014 FasL gene‐844T/C mutation of esophageal cancer in South China and its clinical significance. Sci. Rep. 4:3866. 24473454 10.1038/srep03866 PMC5379236

[29] Sun, T. , X. Miao , X. Zhang , W. Tan , P. Xiong , and D. Lin . 2004 Polymorphisms of death pathway genes FAS and FASL in esophageal squamous‐cell carcinoma. J. Natl Cancer Inst. 96:1030–1036. 15240787 10.1093/jnci/djh187

[30] Jain, M. , S. Kumar , P. Lal , A. Tiwari , U. C. Ghoshal , and B. Mittal . 2007 Role of BCL2 (ala43thr), CCND1 (G870A) and FAS (A‐670G) polymorphisms in modulating the risk of developing esophageal cancer. Cancer Detect. Prev. 3:225–232. 17561354 10.1016/j.cdp.2007.04.005

[31] Chen, X. 2009 Genetic polymorphisms in STK15 and MMP‐2 associated susceptibility to esophageal cancer in Mongolian population. Zhonghua yu fang yi xue za zhi [Chinese J. Prev. Med.] 43:559–564. 19954064

[32] Kim, H. J. , X. M. Jin , H. N. Kim , I. K. Lee , K. S. Park , M. R. Park , et al. 2010 Fas and FasL polymorphisms are not associated with acute myeloid leukemia risk in Koreans. DNA Cell Biol. 29:619–624. 20438363 10.1089/dna.2010.1032

[33] Kupcinskas, J. , T. Wex , J. Bornschein , M. Selgrad , M. Leja , E. Juozaityte , et al. 2011 Lack of association between gene polymorphisms of Angiotensin converting enzyme, Nod‐like receptor 1, Toll‐like receptor 4, FAS/FASL and the presence of Helicobacter pylori‐induced premalignant gastric lesions and gastric cancer in Caucasians. BMC Med. Genet. 12:112. 21864388 10.1186/1471-2350-12-112 PMC3166912

