# Supplementary material for: Quantitative assessment of the association between Fas/FasL gene polymorphism and susceptibility to esophageal carcinoma in a north Chinese population
Source: Cancer Med. 2016 Jan 27;5(4):760–6. doi: 10.1002/cam4.633 (PMC4831295; doi:10.1002/cam4.633)
Supplement: Supplementary file 1 — Table S1. Supplementary Table 1 PCR and RFLP procedures and expected products of three polymorphisms in Fas/FasL gene. [file CAM4-5-760-s001.doc]

| **Supplementary Table 1 PCR and RFLP procedures and expected products of three polymorphisms in Fas/FasL gene** | | | | |
| --- | --- | --- | --- | --- |
| **Reference SNP** | **Primers (forward and reverse)** | **PCR products** | **Restriction enzymes** | **Restriction products** |
| Fas-670 A/G | F:5’-ATAGCTGGGGCTATGCGATT-3’  R:5’-CATTTGACTGGGCTGTCCAT-3’ | 193bp | ScrFI (37℃) | AA:193bp |
|  |  |  |  | GG:136bp,57bp |
|  |  |  |  | AG:193bp,136bp,57bp |
| Fas-1377 G/A | F:5’-TGTGTGCACAAGGCTGGCGC-3’  R:5’-TGCATCTGTCACTGCACTTACCACCA-3’ | 122bp | BstUI (60℃) | AA:122bp |
|  |  |  |  | GG:104bp,18bp |
|  |  |  |  | AG:122bp,104bp,18bp |
| FasL-844 T/C | F:5’-CAGCTACTCGGAGGCCAAG-3’  R:5’-GCTCTGAGGGGAGAGACCAT-3’ | 401bp | BsrDI (65℃) | AA:401bp |
|  |  |  |  | GG:233bp,168bp |
|  |  |  |  | AG:401bp,233bp,168bp |
| ^*^ F and R indicate forward and reverse primers, respectively. | | | | |
